# Modifications in Electrocardiographic and Vectordardiographic Morphological Parameters in Elderly Males as Result of Cardiovascular Diseases and Diabetes Mellitus

**DOI:** 10.3390/diagnostics12122911

**Published:** 2022-11-23

**Authors:** Giovanni Bortolan, Ivaylo Christov, Iana Simova

**Affiliations:** 1 Institute of Neuroscience-National Research Council, IN-CNR, 35127 Padova, Italy; 2 Institute of Biophysics and Biomedical Engineering, Bulgarian Academy of Sciences, 1113 Sofia, Bulgaria; 3 Heart and Brain Center of Excellence, University Hospital Pleven, 5804 Pleven, Bulgaria

**Keywords:** computerized electrocardiography, elderly males, risk of cardiac death, gender medicine

## Abstract

Purpose. Morphological electrocardiographic and vectorcardiographic features have been used in the detection of cardiovascular diseases and prediction of the risk of cardiac death for a long time. The objective of the current study was to investigate the morphological electrocardiographic modifications in the presence of cardiovascular diseases and diabetes mellitus in an elderly male population, most of them with multiple comorbidities. Methods. A database of ECG recordings from the Italian Longitudinal Study on Aging (ILSA-CNR), created to evaluate physiological and pathological modifications related to aging, was considered. The study examined a group of 1109 males with full clinical documentation aged 65–84 years. A healthy control group (219 individuals) was compared to the groups of diabetes mellitus (130), angina pectoris (99), hypertension (607), myocardial infarction (160), arrhythmia (386), congestive heart failure (73), and peripheral artery disease (95). Twenty-one electrocardiographic features were explored, and the effects of cardiovascular diseases and diabetes on these parameters were analyzed. The three-years mortality index was derived and analyzed. Results and Conclusions. Myocardial infarction and arrhythmia were the diagnostic groups that showed a significant deviation of 11 electrocardiographic parameters compared to the healthy group, followed by hypertension and congestive heart failure (10), angina pectoris (9), and diabetes mellitus and peripheral artery disease (8). In particular, a set of three parameters (QRS and T roundness and principal component analysis of T wave) increased significantly, whereas four parameters (T amplitude, T maximal vector, T vector ratio, and T wave area dispersion) decreased significantly in all cardiovascular diseases and diabetes mellitus with respect to healthy group. The QRS parameters show a more specific discrimination with a single disease or a group of diseases, whereas the T-wave features seems to be influenced by all the pathological conditions. The present investigation of disease-related electrocardiographic parameters changes can be used in assessing the risk analysis of cardiac death, and gender medicine.

## 1. Introduction

Morphological electrocardiographic (ECG) and vectorcardiographic (VCG) features have been used for a long time in the identification of cardiovascular diseases and risk analysis. For example, the ST-segment elevation was considered as a risk factor in various groups of haemodialysis (HD) subjects, [1,2]. Prolonged QRS was associated with a significant increase in mortality or sudden death [3,4]. Several studies, including [5,6], showed increased values of QT-interval dispersion (QTd) in several cardiac diseases. The spatial QRS-T angle emerged to be a significant predictor of cardiovascular mortality in a general medical population of 46,573 patients followed for six years [7]. QRS-, and T-amplitude changes of ECG recordings, taken in an interval of three years, significantly decreased with advancing age, in males, but not in females [8]. QRS morphology parameters based on principal component analysis (PCA) was considered during diagnostic pharmacological (Ajmaline) test for suspected Brugada syndrome [9]. Interesting results were given by a recent ECG repolarization parameter, T-wave area dispersion (TWAD), defined by the average of T-wave areas normalized by the maximum absolute area in a set of standard leads [10]. Low TWAD values were a powerful predictor of sudden cardiac death (SCD) during the follow-up of two epidemiological studies. TWAD was measured in pre-, and postoperative ECG recordings of coronary artery bypass grafting (CABG) patients [11]. In preoperative patients, TWAD was lower in diabetics than in nondiabetics showing higher risk of SCD in diabetics. In first 10 postoperative days TWAD decreased proportionally, indicating increased cardiac risk in both groups. An attempt to summarize the influence of various heart diseases on changes in ECG parameters’ set, with a special accent to coronary artery bypass grafting, was performed in [12], and the initial part of the study was roughly presented in [13]. The objective of the current study was to investigate and characterize the impact of morphological ECG and VCG modifications due to cardiovascular diseases and diabetes mellitus in elderly male population where most of them were affected by multi-morbidity. In particular, after the establishment of a consistent group of patients that can characterize a general population of elderly males, the statistical analysis of 10 ECG parameters, 10 VCG parameters and heart rate, obtained by computerized electrocardiography, will put in evidence the significant modifications as a result of the presence of cardiovascular diseases and diabetes mellitus. The present investigation of disease-related electrocardiographic parameters changes can be used for risk analysis of cardiac death, and gender medicine.

## 2. Materials and Methods

The participants to this study were drawn from the Italian Longitudinal Study on Aging (ILSA-CNR), a cross-sectional general population based epidemiological survey, created to evaluate physiological and pathological modifications related to aging [8,14,15,16,17] (see Appendix A for the list of the ILSA Working Group).

The data acquisition was performed at baseline (t1) and after three years (t2). The database consisted of a random sample of individuals aged 65–84 years, living independently or in institutions, stratified by age and sex by using the equal allocation strategy, identified on the demographic list of the registry office of eight Italian municipalities: Genova, Segrate (Milan), Selvazzano-Rubano (Padova), Impruneta (Florence), Fermo (Ascoli Piceno), Naples, Casamassima (Bari), and Catania. The first set of standard 12-leads ECGs was acquired between 1992 and 1993, and the second between 1995 and 1996, when the three-year mortality was derived. All the individuals underwent standardized clinical investigation by a specialist for the final decision on the diagnosis. A detailed description of the characteristics of the diseases was extensively reported in [14].

In this work, a subset of 1109 male patients was considered, and four groups were identified according to their baseline medical classification [8,14,15]:

1. Subjects with a history of cardiovascular disease: arrhythmia (ARRH), angina pectoris (AP), myocardial infarction (MI), congestive heart failure (CHF), hypertension (HTN), peripheral artery disease (PAD);
2. Subjects with diabetes mellitus DM;
3. Subjects with neurological conditions: dementia of any type, distal symmetric neuropathy of lower limbs, parkinsonism, stroke; and
4. Healthy subjects characterized by the absence of any cardiovascular, neurological, chronic pulmonary disease or diabetes, by the absence of therapy potentially influencing cardiac electrical activity, and by the absence of electrolyte imbalance.

Participants in the current study were males, because our preliminary investigation [8] had shown a diminished stratifying ability in the all-gender group, due to male/female opposite biases. Clinical information regarding male participants (n = 1109) was presented in Table 1. The neurological group (group 3), not relevant to the current study, was not reported.

The acquisition of standard twelve-lead ECG recordings was performed for an interval of 10 sec at the frequency of 500 Hz. The ECG signals were preprocessed to eliminate or suppress the powerline interference [18], the drift of the baseline [19] and the electromyographic noise [20]. QRS detection was applied [21], and the identification of the corresponding fiducial points reported in [22] were computed, including the identification of T-wave-end defined in [23]. The precise delineation of Q and T fiducial points and the exact measurements of QT interval were validated under the PhysioNet/Computers in Cardiology Challenge 2006 [24]. Mean P-QRS-T interval was formed by best matching (cross-correlation) of successive P-QRS-T intervals. The use of a mean signal excluded the random selection of an atypical or noise-contaminated signal. Ectopic beats were automatically recognized and were excluded from the mean P-QRS-T. Fiducial points, such as the beginning and end of the P-, QRS-, and T-waves, were allocated automatically on the mean P-QRS-T interval, using algorithms presented in [22,23,24]. Only ECGs at the time of the first recording t1 were used in the study. ECGs at the time of the second t2 = t1 + 3 years were not considered because the effect of aging would bias the results and would be a subject of a further study. Orthogonal Frank X, Y, and Z leads (VCG) were derived from the standard 12-leads by using the transfer matrix of Dower, 1968 [25]. Several vectorcardiographic 3-D and frontal plane 2-D parameters were measured such as maximal vector amplitude of QRS- and T-loops, area of the loops, and angles of the maximal vectors.

For this study, 21 parameters were considered: 10 from ECG, 10 from VCG and the heart rate (HR). All electrocardiographic and vectorcardiographic parameters were automatically measured [26], thus assuring repeatability of the results and lack of intuitive subjectivism, typical to the manual marking and measurement. The considered electrocardiographic parameters were listed in the following, with a brief description of the nonstandard features:

- QRS-amplitude, QRS-PCA, QRS-duration, QT-interval duration, T duration, QT-dispersion, ST-elevation, T-amplitude, T-PCA, and T-area dispersion computed from ECG signal;
- QRS-area, maximal QRS-vector (QRS-max_vect), QRS-angle, QRS-roundness index, T-area, maximal T-vector (T-max_vect), T-angle, QRS/T-angle, T-vector ratio, and T- roundness index computed from VCG signal;
- Heart rate.

QRS-amplitude (peak-peak), T amplitude and ST-elevation was measured in lead V2. QRS-PCA and T-PCA was computed by PCA through singular value decomposition, applied to QRS and T-wave intervals of all 12 ECG leads. Usually, the first three components of the eigenvectors provided the total variation/information of the original data, and the first component gave the greatest contribution. For this study, the ratio between the second and the first eigenvalues (complexity index) was used:

QRS-PCA = λ_2(QRS)_/λ_1(QRS)_

T-PCA = λ_2(T)_/λ_1(T)_.

T-area dispersion TWAD [11] was calculated as the average of T-wave areas in a group of standard leads, normalized by their maximum absolute area:

$$\mathrm{TWAD}=\frac{1}{N}\sum_{i=1}^{N}\frac{Area_{i}\;}{\max(\left|Area_{i:N}\right|}i\in \{I,\;\mathrm{II},\;V_{4},\;V_{5},V_{6}\}.$$

**Figure 1:**
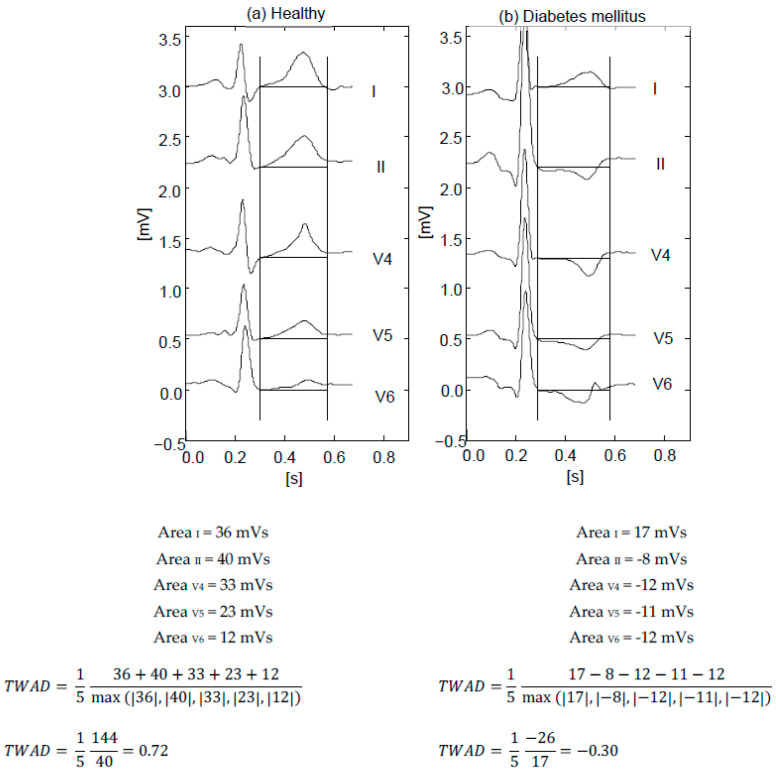
TWAD in a healthy patient (**a**) and in a patient with diabetes mellitus (**b**).

TWAD was calculated from leads {I, II, V_4_, V_5_, V_6_}, because T waves in these leads were normally positive, and inversions in them conveyed prognostic value. TWAD values were between −1 (all identical negative areas) and +1 (identical positive areas). An example of TWAD calculation was shown in Figure 1.

In X-Y-Z leads, the magnitude of the vectors V for sample “n” were computed by

$$Vn_{\;}=\sqrt{X_{n}^{2}+Y_{n}^{2}+Z_{n}^{2}}.$$

The T-loop vector ratio (VR_T_) was defined by [27].

T-vector ratio = max(V_n_)/mean(V_n_) for {n} in T loop and it was considered in [28,29] for the characterization of T-loop morphology with some cardiovascular diseases.

The roundness index (RI) for QRS and T waves, measured as the ratio of the area of the QRS or T loop to the square of the corresponding maximum vector [30], characterizes the circular shape of the 3-D loop:

RI_QRS_ = QRS Area/maxV_n_^2^    for n in QRS loop

RI_T_ = T Area/maxV_n_^2^        for n in T loop.

**Figure 2:**
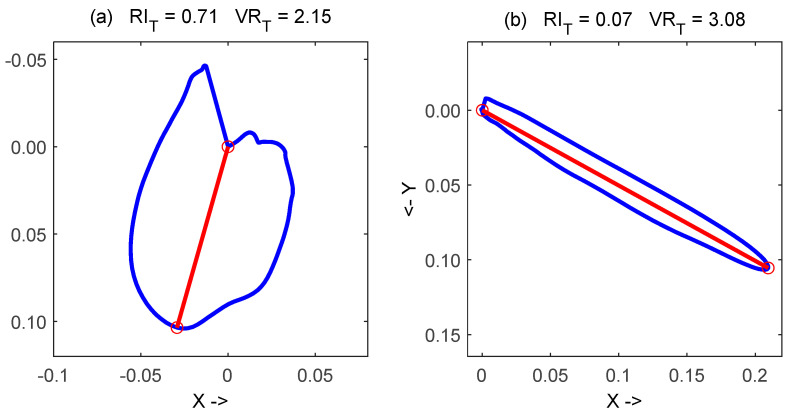
Roundness of T-loop, expressed by roundness index (RI_T_) and T-vector_ratio (VR_T_) in a male patient with myocardial infarction and congestive heart failure (**a**) and in healthy male (**b**).

The healthy/nonhealthy differentiation ability of the roundness index (RI_T_) and T-vector ratio (VR_T_) was illustrated in Figure 2, where the two indices were reported for a healthy male (right) and for a male with myocardial infarction and congestive heart failure (left).

The parameters of the considered groups were described by descriptive statistics (mean and standard deviation) and by the limits of the 95% confidence interval. Nonparametric Wilcoxon rank sum test (for equality of population medians) was used for testing the statistical significance of the comparisons between healthy groups vs. groups with cardiac diseases and diabetics. All statistical analysis and data visualization were performed with Matlab V.9.7 (© 1994–2022 The MathWorks, Inc., Natick, MA, USA; www.mathworks.com).

## 3. Results

All the electrocardiographic and vectorcardiographic features were automatically measured, and the statistical analysis was performed for all the eight diagnostic groups of the ILSA database. The mean values, the standard deviation and the 95% confidence interval of all considered 21 parameters were reported in Table 2. A comparison between the healthy group and all the other diagnostic groups was performed in order to test and to quantify their influence on the electrocardiographic parameters. In particular, the parameters of the healthy control group (219 individuals) were compared with respect to the other groups: diabetics (DM) (130), angina pectoris (AP) (99), hypertension (HTN) (607), myocardial infarction (MI) (160), arrhythmia (ARRH) (386), congestive heart failure (CHF) (73), and peripheral arterial diseases (PAD) (95). Patients with two or more diseases (see Table 1) were examined more than ones in the corresponding groups. The results of this comparison were presented in Table 3. Statistically significant results (*p* < 0.01) were reported in this Table.

All heart diseases and diabetes mellitus lead to monotonic significant changes in the morphological electrocardiographic features with respect to healthy individuals. It was interesting to compare the behaviour of the parameters connected to QRS wave or T-wave. The seven parameters related to QRS-complex were tested 49 times. Statistically significant differences were obtained in 18 comparisons (37%). The nine parameters related to T-wave were tested 63 times. Statistically significant results were obtained 43 times (68%). This was a clear indication that T-wave-related parameters have a higher discriminant power with respect to the QRS ones. Another interesting result was given by the fact that among the nine parameters related to T wave, six presented significant differences compared to the healthy group for all the seven diagnostic groups, and for two parameters no significant differences were found. On the other hand, none of the seven parameters related to QRS waves presented a significant difference with all diseases, but only three parameters presented differences with five or six diagnostic groups. This probably means that the QRS was more specific for diseases, while the T wave modifications were more general.

It was also possible to analyse the behavior of the 10 parameters extracted from VCG and 10 from ECG. In this case it turns out there was no statistical difference between the two sets of parameters (significant differences in 52.5% for VCG parameters and 55% for ECG) confirming an equivalent discriminant power in the differentiation of healthy controls with respect to nonhealthy groups. All these remarks were evident observing the Table 3.

Table 4 reports the three-year mortality percentage of the different diagnostic groups. As expected, healthy group has lower mortality (3.2%), whereas the highest value was obtained by the congestive heart failure group (23.3%). All the remaining groups lies in the range [8.4%, 12.5%].

A subsequent comparison between the deceased and living after three years considering all the diagnostic groups and all the morphological electrocardiographic features were reported in Table 5. Parameters with a significant statistical difference between alive and deceased people in three years were reported in the table. In the healthy group, two parameters connected to the heterogeneity of repolarization phase (QT dispersion and T wave dispersion) presented significant differences in Table 5. Both the differences were in agreement with the literature: living people presented higher values of TWAD and lower values of QT dispersion. In addition, the group with highest mortality index (CHF) was the only one with no statistical differences. Significant changes were more frequent in T-wave related parameters (16.3%) than in QRS-complex related ones (3.5%). We have to notice that the size of the population was quite limited for a mortality discussion.

## 4. Discussion

Myocardial infarction and arrhythmia were the two diagnostic groups that showed a significant deviation of 11 electrocardiographic parameters from the healthy group, 10 significant deviations for hypertension and congestive heart failure groups, nine for angina pectoris group, while eight parameters differ significantly in peripheral artery disease and diabetes mellitus groups. A detailed list of the main differences will follow.

The myocardial infarction showed the following significant differences with respect to the healthy control group:

- Increased values for area of QRS-loop (*p* = 0.005), QRS duration (*p* < 0.001), QT-dispersion (*p* = 0.006), T-wave PCA (*p* < 0.001), T-wave roundness index (*p* < 0.001), and QRS-roundness index (*p* < 0.001) (the loop resembles more a circle than a narrow ellipsoid); and
- Decreased values for maximal vector of QRS-loop (*p* < 0.001), T amplitude (*p* < 0.001), maximal vector of T-loop (*p* < 0.001), and T-wave area dispersion (*p* < 0.001), and T-loop vector ratio (*p* < 0.001) (the shape of the loop has become more oval or circular);

The arrhythmia group showed the same significant deviations like the MI group except for QRS max vector (n.s.) and HR (*p* = 0.003).

The hypertension group showed the following significant deviation:

- Increased values for QRS amplitude (*p* = 0.008), area of QRS-loop (*p* < 0.001), QRS-duration, (*p* < 0.001), QRS-roundness index (*p* < 0.001), T-wave PCA (*p* < 0.001), T-wave roundness index (*p* < 0.001);
- Decreased values for T-wave amplitude (*p* < 0.001), maximal vector of T-loop (*p* < 0.001), T-loop vector ratio (*p* < 0.001), and T-wave area dispersion (*p* < 0.001);

The angina pectoris group showed a significant deviation with the following parameters:

- Increased values for area of QRS-loop (*p* = 0.007), QRS-roundness index (*p* = 0.002), QT-duration (*p* = 0.006), T-duration (*p* = 0.045), T-wave PCA (*p* < 0.001), T-wave roundness index (*p* < 0.001); and
- Decreased values for: T-wave amplitude (*p* < 0.001), magnitude of the maximal vector of T-loop (*p* < 0.001), T-loop vector ratio (*p* < 0.001), and T-wave area dispersion (*p* < 0.001).

CHF, the group with highest three-year mortality index, has the highest values for T roundness and lowest values for T-vector_ratio and T_PCA, showing more circular shape of T-loop vector, and lowest and negative mean values of TWAD indicative for presence of negative T-wave in leads I, II, V4, V5, V6.

It will follow a descriptive analysis of the behaviour of the considered morphological electrocardiographic features in the comparison of the cardiovascular diseases and diabetes mellitus with respect to healthy control.

- QRS-amplitude increase was significant only for the HTN group. This was in accordance with [31], where increase of R and S-peaks in the group of HTN compared to the group of normotensive, was found in the examined leads;
- The area of QRS-loop increased significantly in AP, HTN, MI, ARRH, and PAD. This parameter was used in patient selection for cardiac resynchronization therapy [32], and showed, like a LBBB index, more predictive power than other electrocardiographic parameters.
- Decrease of maximal vector of QRS-loop was statistically significant only for MI. This result was in agreement with [30] where QRS vector magnitude decreased significantly in patients with infarct-cardiomyopathy and ventricular tachycardia with respect to healthy control subjects.
- The analysis of QRS and T-wave by means of PCA was performed in order to find a quantitative expression of the morphological changes of those waves. The QRS-PCA contributed to the diagnosis and risk stratification of patients with Brugada syndrome [9] and PCA of the T-wave was used for the detection of T-wave alternans (TWA) [33]. In our study, only T-wave PCA presented significant differences and increased in all cardiovascular diseases and DM compared to the healthy group.
- The QRS-duration was a well-known prognostic factor [3,4], and this was confirmed in the current study, in which a significant increase of the median values of 2 to 6 ms were obtained in DM and all cardiovascular diseases, except AP and PAD;
- The QRS roundness index resulted in significant increase in all the considered diseases. This index was studied in literature [30,34] with contrasting results mainly for the limited number of examined subjects;
- QT-interval duration increased significantly only in AP;
- The QT-dispersion index increased significantly in MI and HTN groups, and this was supported by [6] wherein increased values of this index were associated with higher risk of cardiac death;
- The T-wave amplitude decreased significantly in all cardiovascular diseases and in DM with respect to the healthy control. This result was supported by a recent study [35], wherein this index was associated as a marker of sudden cardiac arrest in the high risk group of patients with hypertrophic cardiomyopathy;
- A significant decrease of the magnitude of T-loop maximal vector was observed for all cardiovascular diseases and for DM. The importance of this parameter in detection of myocardial injury after infarction was pointed out in [36];
- The observed significant decrease of T vector ratio and significant increase of T roundness index in all considered diseases, with respect to healthy subjects, has a very interesting visual interpretation: the T-loop in these patients developed a rounder shape, like the examples reported in Figure 2;
- T-wave area dispersion was a parameter recently proposed for quantification of repolarization heterogeneity, and low TWAD values were associated with SCD during a follow-up of general population [10]. In addition, TWAD was used to identify successfully DM patients as a group with increased risk of SCD at the time of coronary artery bypass grafting [11]. In the current study, TWAD decreased significantly for all cardiovascular diseases and for DM, and reached the lowest values (negative) for CHF. This shows the higher discriminant power of TWAD with respect to QT-dispersion for characterization of the repolarization phase in ECG. In addition, the simpler computation of TWAD should be pointed out, in which the T-wave localization was not so critical as in QT-wave dispersion [11].
- Heart rate increased significantly in DM and in all cardiovascular diseases except in AP group. This result was in agreement with several studies in literature, and the HR was consequently considered as a risk factor for various cardiac events [37]. In [38], HR increased significantly after heart operation, and it was influenced by many factors from cardiac and hemodynamic origin but also from general factors such as pain, hypoxia, or hypothermia.

## 5. Conclusions

The main aim of this study was to investigate how cardiovascular diseases and diabetes mellitus impact 21 electrocardiographic and vectorcardiographic parameters with respect to healthy control group, considering an elderly male population, most of whom had multi-morbidities.

Myocardial infarction and arrhythmia were the diseases that influenced the highest number of electrocardiographic parameters, followed by hypertension and peripheral artery disease. There was a different behaviour of QRS and T-wave parameters in the discrimination process between healthy control and the seven diagnostic groups. Six parameters were able to discriminate all cardiovascular diseases and DM with respect to the healthy. They all were from T-wave analysis (T-roundness index, T-max_vector, T-vector ratio, T-amplitude, PCA_T, and T-wave area dispersion). The QRS parameters show a more specific discrimination with single or group of diseases, whereas the T-wave features seems to be influenced by (all) the pathological conditions. In particular, QRS-area, QRS-duration, and QRS-roundness presented increasing values all in three specific diseases (HTN, MI, and ARRH). From a statistical point of view, significant changes due to cardiac diseases or DM were more frequent in T-wave related parameters than in QRS-complex related ones. All heart diseases and diabetes mellitus lead to monotonic significant changes (all increased values or all decreased values) in the morphological electrocardiographic features with respect to healthy. The present investigation of disease-related electrocardiographic parameters changes can be used in assessing the risk analysis of cardiac death, and gender medicine.

The present work has several limitations, for example the relatively small size of some single groups of diseases, and the presence of comorbidity may have some hidden connections and effects, and it may affect the statistical analysis. Another limitation is given by the mortality analysis performed without the use of the precise time of death across the three-year follow-up, not permitting univariate and multivariate risk analysis, which is postponed to a future work. In addition, the choice of analyzing only the male population is simultaneously a characterizing point and a limitation. For this reason, the next step is to perform a similar analysis with the female population, in order to check similarities and differences between male and female groups.

## Figures and Tables

**Table 1:** Clinical characteristics of the males’ group of ILSA database. (AP, angina pectoris; ARRH, arrhythmia; CHF, congestive heart failure; DM, diabetes mellitus; HTN, hypertension; MI, myocardial infarction; PAD, peripheral artery disease).

	Healthy	DM	AP	HTN	MI	ARRH	CHF	PAD
Healthy	219	0	0	0	0	0	0	0
DM.	0	130	14	96	28	57	10	20
AP	0	14	99	69	41	41	15	17
HTN	0	96	69	607	99	252	50	69
MI	0	28	41	99	160	72	28	26
ARRH	0	57	41	252	72	386	47	40
CHF	0	10	15	50	28	47	73	9
PAD	0	20	17	69	26	40	9	95

**Table 2:** Mean values (standard deviation) and 95% confidence interval of the 21 electrocardiographic and vectorcardiographic features, with the corresponding units, in males for cardiovascular diseases and diabetes mellitus and healthy control group.

	DM (n = 130)	AP (n = 99)	HTN (n = 607)	MI (n = 160)	ARRH (n = 386)	CHF (n = 73)	PAD (n = 95)	Healty (n = 219)
QRS-ampl [mV]	0.83 (0.39) [0.76; 0.90]	0.86 (0.33) [0.80; 0.93]	0.90 (0.38) [0.87; 0.93]	0.76 (0.33) [0.71; 0.82]	0.86 (0.39) [0.82; 0.90]	0.84 (0.48) [0.72; 0.95]	0.80 (0.34) [0.73; 0.87]	0.82 (0.31) [0.78; 0.86]
QRS-area [mV*s]	0.14 (0.15) [0.12; 0.17]	0.18 (0.24) [0.13; 0.23]	0.16 (0.18) [0.14; 0.17]	0.16 (0.16) [0.13; 0.18]	0.17 (0.22) [0.15; 0.19]	0.18 (0.29) [0.12; 0.25]	0.16 (0.14) [0.13; 0.19]	0.11 (0.11) [0.10; 0.13]
QRS-max_vect [mV]	0.77 (0.31) [0.72; 0.82]	0.79 (0.33) [0.72; 0.86]	0.80 (0.30) [0.78; 0.82]	0.68 (0.27) [0.64; 0.72]	0.77 (0.30) [0.74; 0.80]	0.77 (0.34) [0.69; 0.85]	0.75 (0.29) [0.69; 0.81]	0.77 (0.25) [0.74; 0.80]
QRS-angle [°]	123.5 (68.1) [111.7; 135.3]	130.0 (75.2) [115.0; 145.0]	121.3 (67.5) [115.9; 126.7]	126.3 (79.0) [114.0; 138.6]	123.1 (74.2) [115.6; 130.5]	122.6 (72.8) [105.6; 139.6]	117.5 (66.5) [104.0; 131.1]	108.6 (41.8) [103.0; 114.2]
QRS-PCA [number]	0.24 (0.21) [0.20; 0.28]	0.21 (0.18) [0.18; 0.25]	0.24 (0.19) [0.22; 0.25]	0.24 (0.21) [0.20; 0.27]	0.24 (0.20) [0.22; 0.26]	0.23 (0.21) [0.18; 0.28]	0.27 (0.22) [0.22; 0.31]	0.26 (0.20) [0.23; 0.29]
QRS-durat [ms]	126.7 (21.4) [123.0; 130.4]	122.6 (21.9) [118.3; 127.0]	123.8 (22.9) [122.0; 125.6]	125.2 (20.8) [121.9; 128.4]	128.3 (27.6) [125.6; 131.1]	128.3 (27.7) [121.8; 134.7]	122.4 (21.3) [118.1; 126.7]	116.8 (14.8) [114.9; 118.8]
QRS-round [number]	0.24 (0.20) [0.20; 0.27]	0.30 (0.25) [0.24; 0.35]	0.26 (0.22) [0.25; 0.28]	0.34 (0.24) [0.30; 0.38]	0.29 (0.23) [0.26; 0.31]	0.29 (0.25) [0.23; 0.34]	0.32 (0.25) [0.27; 0.37]	0.22 (0.21) [0.19; 0.24]
QT-durat [ms]	426.9 (39.6) [420.0; 433.7]	432.6 (48.4) [422.9; 442.2]	427.7 (43.5) [424.3; 431.2]	430.2 (41.9) [423.7; 436.7]	428.5 (49.9) [423.5; 433.5]	415.3 (50.7) [403.5; 427.1]	429.4 (47.4) [419.7; 439.1]	422.5 (37.0) [417.6; 427.4]
QT-disp [ms]	41.04 (12.48) [38.87; 43.20]	41.16 (13.00) [38.56; 43.75]	40.13 (12.00) [39.17; 41.09]	41.41 (11.61) [39.60; 43.23]	41.47 (11.92) [40.27; 42.66]	40.91 (13.19) [37.83; 43.99]	40.01 (11.61) [37.65; 42.38]	37.74 (12.32) [36.10; 39.38]
ST-elevat [mV]	77.62 (77.08) [64.24; 90.99]	86.16 (253.73) [35.55; 136.76]	74.97 (119.49) [65.45; 84.50]	75.08 (82.13) [62.26; 87.91]	82.54 (227.94) [59.73; 105.35]	50.00 (104.46) [25.63; 74.37]	56.21 (80.46) [39.82; 72.60]	69.67 (54.46) [62.41; 76.92]
T-duration [ms]	300.2 (38.3) [293.5; 306.8]	309.9 (43.1) [301.3; 318.5]	303.9 (40.5) [300.7; 307.2]	305.1 (38.0) [299.1; 311.0]	300.2 (45.0) [295.7; 304.7]	287.0 (46.9) [276.1; 298.0]	307.0 (42.1) [298.4; 315.6]	305.7 (38.2) [300.6; 310.8]
T-ampl [mV]	0.08 (0.07) [0.07; 0.10]	0.08 (0.07) [0.07; 0.10]	0.11 (0.09) [0.10; 0.11]	0.08 (0.07) [0.07; 0.09]	0.10 (0.08) [0.09; 0.10]	0.08 (0.07) [0.06; 0.09]	0.09 (0.07) [0.08; 0.11]	0.14 (0.08) [0.13; 0.15]
T-area [mV*s]	5.32 (6.13) [4.26; 6.38]	6.59 (7.37) [5.12; 8.06]	6.79 (11.27) [5.89; 7.68]	6.10 (8.80) [4.73; 7.47]	7.04 (13.61) [5.68; 8.41]	9.84 (26.84) [3.58; 16.11]	7.02 (8.27) [5.34; 8.71]	6.12 (6.18) [5.30; 6.95]
T-max_vect [mV]	0.20 (0.11) [0.18; 0.22]	0.19 (0.09) [0.18; 0.21]	0.23 (0.11) [0.22; 0.24]	0.19 (0.11) [0.17; 0.21]	0.22 (0.11) [0.21; 0.23]	0.19 (0.10) [0.17; 0.21]	0.20 (0.11) [0.18; 0.23]	0.26 (0.10) [0.25; 0.27]
T-angle [°]	108.5 (33.6) [102.7; 114.4]	116.5 (33.8) [109.8; 123.2]	113.4 (31.0) [111.0; 115.9]	115.8 (47.1) [108.5; 123.2]	115.2 (36.9) [111.5; 118.9]	120.4 (45.0) [109.9; 130.9]	111.0 (28.9) [105.1; 116.9]	111.1 (12.9) [109.4; 112.8]
QRS/T-angle [°]	−14.95 (73.45) [−27.70; −2.20]	−13.51 (79.54) [−29.37; 2.35]	−7.87 (72.05) [−13.61; −2.13]	−10.48 (85.11) [−23.77; 2.80]	−7.87 (81.58) [−16.03; 0.30]	−2.22 (85.35) [−22.13; 17.70]	−6.53 (72.74) [−21.35; 8.28]	2.53 (42.83) [−3.18; 8.23]
T-vector ratio [number]	2.38 (0.44) [2.30; 2.45]	2.41 (0.47) [2.32; 2.51]	2.45 (0.43) [2.41; 2.48]	2.34 (0.46) [2.27; 2.41]	2.35 (0.45) [2.30; 2.39]	2.29 (0.42) [2.19; 2.39]	2.36 (0.45) [2.27; 2.46]	2.64 (0.39) [2.59; 2.69]
T-PCA [number]	0.13 (0.13) [0.11; 0.15]	0.14 (0.15) [0.11; 0.17]	0.12 (0.13) [0.11; 0.13]	0.13 (0.14) [0.11; 0.15]	0.13 (0.14) [0.11; 0.14]	0.18 (0.17) [0.14; 0.22]	0.13 (0.13) [0.10; 0.16]	0.07 (0.07) [0.06; 0.08]
T-roundness [number]	0.17 (0.18) [0.14; 0.20]	0.18 (0.20) [0.14; 0.22]	0.15 (0.16) [0.14; 0.16]	0.19 (0.20) [0.16; 0.23]	0.16 (0.18) [0.15; 0.18]	0.21 (0.22) [0.15; 0.26]	0.20 (0.22) [0.16; 0.25]	0.09 (0.07) [0.08; 0.10]
TWAD [number]	0.09 (0.44) [0.02; 0.17]	0.09 (0.45) [−0.00; 0.18]	0.19 (0.42) [0.16; 0.23]	0.09 (0.44) [0.02; 0.16]	0.16 (0.44) [0.12; 0.20]	−0.05 (0.44) [−0.15; 0.06]	0.16 (0.41) [0.08; 0.24]	0.38 (0.33) [0.34; 0.43]
HR [bpm]	69.97 (12.01) [67.88; 72.05]	67.41 (13.25) [64.77; 70.06]	68.53 (11.96) [67.57; 69.48]	68.90 (11.24) [67.15; 70.65]	70.22 (14.18) [68.80; 71.63]	76.04 (15.96) [72.32; 79.77]	69.60 (12.05) [67.15; 72.05]	66.11 (9.89) [64.79; 67.42]

**Table 3:** Comparison of healthy vs. cardiovascular diseases and diabetes groups (significant *p*-values *p* < 0.01 were reported) considering 21 electrocardiographic and vectorcardiographic parameters.

	Healthy vs:						
	DM (n = 130)	AP (n = 99)	HTN (n = 607)	MI (n = 160)	ARRH (n = 386)	CHF (n = 73)	PAD (n = 95)
QRS-ampl			*p* = 0.008				
QRS-area		*p* = 0.007	*p* < 0.001	*p* = 0.005	*p* < 0.001		*p* = 0.001
QRS-max_vect				*p* < 0.001			
QRS-angle							
QRS-PCA							
QRS-durat	*p* < 0.001		*p* < 0.001	*p* < 0.001	*p* < 0.001	*p* = 0.005	
QRS-round		*p* = 0.002	*p* < 0.001	*p* < 0.001	*p* < 0.001	*p* = 0.005	*p* < 0.001
QT-durat		*p* = 0.006					
QT-disp				*p* = 0.006	*p* < 0.001		
ST-elevat							
T-duration						*p* = 0.003	
T-ampl	*p* < 0.001	*p* < 0.001	*p* < 0.001	*p* < 0.001	*p* < 0.001	*p* < 0.001	*p* < 0.001
T-area							
T-max_vect	*p* < 0.001	*p* < 0.001	*p* < 0.001	*p* < 0.001	*p* < 0.001	*p* < 0.001	*p* < 0.001
T-angle							
QRS/T-angle							
T-vector ratio	*p* < 0.001	*p* < 0.001	*p* < 0.001	*p* < 0.001	*p* < 0.001	*p* < 0.001	*p* < 0.001
T-PCA	*p* < 0.001	*p* < 0.001	*p* < 0.001	*p* < 0.001	*p* < 0.001	*p* < 0.001	*p* < 0.001
T-roundness	*p* < 0.001	*p* < 0.001	*p* < 0.001	*p* < 0.001	*p* < 0.001	*p* < 0.001	*p* < 0.001
TWAD	*p* < 0.001	*p* < 0.001	*p* < 0.001	*p* < 0.001	*p* < 0.001	*p* < 0.001	*p* < 0.001
HR	*p* = 0.003				*p* = 0.003	*p* < 0.001	

**Table 4:** Three-year mortality in the cardiovascular diseases and diabetes groups.

	Healthy	DM	AP	HTN	MI	ARRH	CHF	PAD
Mortality	3.2%	12.3%	11.1%	8.4%	12.5%	10.1%	23.2%	13.7%

**Table 5:** Comparison of the three-year mortality group with respect to the living group for all cardiovascular and diabetic groups, considering the morphological electrocardiographic and vectorcardiographic features (only the significant *p*-values *p* < 0.05 were reported).

	Healthy	DM	AP	HTN	MI	ARRH	CHF	PAD
QRS-ampl								
QRS-area								
QRS-max_vect								
QRS-angle								*p* = 0.033
QRS-PCA			*p* = 0.044					
QRS-durat								
QRS-round								
QT-durat								
QT-disp	*p* = 0.017							
ST-elevat				*p* = 0.034				
T-duration								
T-ampl		*p* = 0.007		*p* = 0.033				
T-area								
T-max_vect		*p* = 0.001		*p* < 0.001				*p* = 0.001
T-angle					*p* = 0.012			
QRS/T-angle								
T-vector_ratio		*p* = 0.029						
T-PCA			*p* = 0.006					
T-roundn		*p* = 0.031		*p* = 0.019		*p* = 0.029		*p* < 0.001
TWAD	*p* = 0.043							
HR					*p* = 0.001			

## Data Availability

Not applicable.

## Appendix A

The ILSA Working Group: E. Scafato, G. Farchi, L. Galluzzo, C. Gandin, Istituto Superiore di Sanitá, Rome; A. Capurso, F. Panza, V. Solfrizzi, V. Lepore, P. Livrea, University of Bari; L. Motta, G. Carnazzo, M. Motta, P. Bentivegna, University of Catania; S. Bonaiuto, G. Cruciani, F. Fini, A. Vesprini, D. Postacchini, Italian National Research Centre on Aging (INRCA), Fermo; D. Inzitari, L. Amaducci, University of Florence; A. Di Carlo, M. Baldereschi, Institute of Neuroscience, National Research Council of Italy (CNR), Florence; A. Ghetti, R. Vergassola, Health Area 10, Florence; C. Gandolfo, M. Conti, University of Genoa; N. Canal, M. Franceschi, San Raffaele Institute, Milan; G. Scarlato, L. Candelise, E. Scarpini, University of Milan; F. Rengo, P. Abete, F. Cacciatore, F. Covelluzzi, University of Naples; G. Enzi, L. Battistin, G. Sergi, G. Crepaldi, M. Bressan, University of Padua; G.Bortolan, National Research Council of Italy (CNR), Padova; S. Maggi, N. Minicuci, M. Noale, National Research Council of Italy (CNR), Aging Section, Padua; F. Grigoletto, E. Perissinotto, Institute of Hygiene, University of Padua; P. Carbonin, Universitá Cattolica del Sacro Cuore, Rome.
